# Supplementary material for: System‐level analyses of keystone genes required for mammalian tooth development
Source: J Exp Zool B Mol Dev Evol. 2020 Oct 31;336(1):7–17. doi: 10.1002/jez.b.23009 (PMC7894285; doi:10.1002/jez.b.23009)
Supplement: Supplementary file 1 — Supporting information. [file JEZ-336-7-s001.docx]

**System-level analyses of keystone genes required for mammalian tooth development**

Outi Hallikas | Rishi Das Roy | Mona M. Christensen | Elodie Renvoisé |

Ana-Marija Sulic | Jukka Jernvall

**Supporting Information:**

**Appendix S1.** Developmental keystone genes of mouse tooth development.

**Table S1.** Genes that are dispensable for mouse tooth development.

**Table S2.** Descriptive statistics of the expression of different gene categories.

**Table S3.** Permutation tests for microarray, RNAseq and scRNAseq expression levels.

**Table S4.** Pathway genes used to compare expression levels.

**Table S5.** Descriptive statistics of the number of cells in which different gene types are expressed.

**Appendix S1.** Developmental keystone genes of mouse tooth development. Classification of genes whose null mutations affect the phenotype of the mouse first lower molar. The classification of each gene is usually based on multiple sources, with key references listed. Haplosufficiency is marked for progression and shape categories.

| Gene name | Mouse Ensembl ID (ENSMUSG) | Category | Haplo-  sufficient | Type of molecule | Mutant tooth phenotype | Ref. |
| --- | --- | --- | --- | --- | --- | --- |
| *Acvr2a* | 00000052155 | progression | Yes | receptor | Bud stage arrest, partial penetrance | 1 |
| *Bmp4* | 00000021835 | progression | Yes? | signal | Wnt1 cKO: Bud or cap stage arrest | 2 3 |
| *Bmpr1a* | 00000021796 | progression | Yes | receptor | K14 cKO: Bud stage arrest | 4 |
| *Ctnna1* | 00000037815 | progression | Yes? | intracellular | K14 cKO: Cap stage arrest | 5 |
| *Ctnnb1* | 00000006932 | progression | Yes? | intracellular | K14 cKO: Early bud stage arrest | 6 |
| *Dicer1* | 00000041415 | progression | Yes? | intracellular | Wnt1 cKO: Arrest at cap stage or absence | 7 8 |
| *Fgfr2* | 00000030849 | progression | Yes | receptor | Bud stage arrest | 9 |
| *Inhba* | 00000041324 | progression | Yes? | signal | Bud stage arrest | 10 11 |
| *Lef1* | 00000027985 | progression | Yes | transcription factor | Late bud stage arrest | 12 13 |
| *Msx1* | 00000048450 | progression | Yes? | transcription factor | Bud stage arrest | 14 |
| *Pax9* | 00000001497 | progression | Yes | transcription factor | Bud stage arrest | 15 |
| *Pitx2* | 00000028023 | progression | Yes | transcription factor | Bud stage arrest | 16 17 |
| *Runx2* | 00000039153 | progression | Yes | transcription factor | Late bud stage arrest, extra budding | 18 19 |
| *Shh* | 00000002633 | progression | Yes | signal | K14 cKO: Cap stage arrest | 20 |
| *Trp63* | 00000022510 | progression | Yes? | transcription factor | Dental placode stage arrest | 21 22 |
| *Apc* | 00000005871 | shape | Yes | intracellular | K14 cKO: Deformed supernumerary teeth | 23 |
| *Barx1* | 00000021381 | shape | Yes | transcription factor | Slightly smaller molars | 24 |
| *Bcl11b* | 00000048251 | shape | Yes? | transcription factor | Blunted cusps and reduced stellate reticuli | 25 26 |
| *Bmp2* | 00000027358 | shape | Yes | signal | Osx cKO: Brittle and slightly misshaped, third molars missing | 27 28 |
| *Bmp7* | 00000008999 | shape | Yes | signal | Abnormal cusp patterning | 29 30 |
| *Chuk* | 00000025199 | shape | Yes | intracellular | Flattened cusps, no third molars | 31 |
| *Eda* | 00000059327 | shape | Yes | signal | Reduced number and size of cusps of the first and second molars | 32 33 34 |
| *Edar* | 00000003227 | shape | Yes | receptor | Reduced teeth | 33 35 |
| *Edaradd* | 00000095105 | shape | Yes | intracellular | Severe agenesis, cone/peg shaped teeth | 36 37 |
| *Evc* | 00000029122 | shape | Yes | intracellular | Conical molars, size reduction of the first molar and an enamel defect | 38 |
| *Fgf10* | 00000021732 | shape | Yes | signal | Smaller molars and/or different shape | 39 40 |
| *Fgf20* | 00000031603 | shape | Yes | signal | Smaller molars with mildly altered cusp pattern | 41 |
| *Fgf3* | 00000031074 | shape | Yes | signal | Change of cusp pattern | 42 |
| *Foxi3* | 00000055874 | shape | Yes | transcription factor | K14 cKO: Misshaped teeth | 43 |
| *Fst* | 00000021765 | shape | Yes? | signal | Non-polarized cusps with multiple shallow foldings | 44 45 |
| *Gas1* | 00000052957 | shape | Yes? | intracellular | Supernumerary teeth, molars slightly misshaped | 46 |
| *Jag2* | 00000002799 | shape | Yes | signal | Increase of cusp number | 47 |
| *Lrp4* | 00000027253 | shape | Yes? | receptor | Deformed supernumerary teeth | 48 49 |
| *Msx2* | 00000021469 | shape | Yes | transcription factor | Msx2 null: Misshaped teeth, enamel hypoplasia | 50 |
| *Pdgfra* | 00000029231 | shape | Yes? | receptor | Slight change in cusp pattern | 51 |
| *Rps6ka3* | 00000031309 | shape | Yes | intracellular | Small change in tooth shape, often additional molars | 52 |
| *Smo* | 00000001761 | shape | Yes | receptor | K14 cKO: Cusp pattern changed, molars fused and reduced | 53 |
| *Sostdc1* | 00000036169 | shape | Yes | signal | Change in cusp pattern, supernumerary teeth | 54 |
| *Sp6* | 00000038560 | shape | Yes? | transcription factor | Supernumerary teeth, reduction of cusp number | 55 |
| *Spry2* | 00000022114 | shape | Yes? | intracellular | Slight change in cusp pattern, supernumerary teeth | 56 |
| *Spry4* | 00000024427 | shape | Yes? | intracellular | Slight change in cusp pattern, supernumerary teeth | 56 |
| *Wnt10a* | 00000026167 | shape | Yes? | signal | Abnormal cusp patterning, smaller and supernumerary teeth | 57 |
| *Yap1* | 00000053110 | shape | Yes? | intracellular | K14 cKO: Smaller and abnormal tooth, fewer and flattened cusps | 58 |
| *Alpl* | 00000028766 | tissue |  | other | Dentinogenesis imperfecta | 59 |
| *Ambn* | 00000029288 | tissue |  | other | Severe enamel hypoplasia, odontogenenic tumors | 60 |
| *Amelx* | 00000031354 | tissue |  | other | Enamel hypoplasia | 61 |
| *Amtn* | 00000029282 | tissue |  | other | Amelogenesis imperfecta | 62 |
| *Bmi1* | 00000026739 | tissue |  | transcription factor | Amylogenesis imperfecta | 63 |
| *Dmp1* | 00000029307 | tissue |  | other | Dentinogenesis imperfecta | 64 65 66 |
| *Dspp* | 00000053268 | tissue |  | other | Dentinogenesis imperfecta | 67 68 |
| *Enam* | 00000029286 | tissue |  | other | No enamel | 69 |
| *Evc2* | 00000050248 | tissue |  | intracellular | Amelogenesis imperfecta | 70 |
| *Fam20a* | 00000020614 | tissue |  | other | Severe amelogenesis imperfecta | 71 |
| *Fam20c* | 00000025854 | tissue |  | other | Severe amelogenesis imperfecta | 71 |
| *Fgfr1* | 00000031565 | tissue |  | receptor | K14 cKO: Amelogenesis imperfecta | 72 |
| *Gdnf* | 00000022144 | tissue |  | signal | Amelogenesis imperfecta, dentinogenesis imperfecta | 73 |
| *Grem2* | 00000050069 | tissue |  | signal | Enamel hypoplasia, dentine hypoplasia | 74 |
| *Klk4* | 00000006948 | tissue |  | other | Amelogenesis imperfecta | 75 |
| *Lama3* | 00000024421 | tissue |  | other | Enamel hypoplasia | 76 |
| *Mmp14* | 00000000957 | tissue |  | other | Amelogenesis imperfecta | 77 78 |
| *Mmp20* | 00000018620 | tissue |  | other | Amelogenesis imperfecta, enamel hypoplasia | 79 |
| *Mtor* | 00000028991 | tissue |  | intracellular | Osx1 cKO: Dentine hypoplasia | 80 |
| *Nectin1* | 00000032012 | tissue |  | other | Amelogenesis imperfecta | 81 81 |
| *Perp* | 00000019851 | tissue |  | other | Amelogenesis imperfecta | 82 |
| *Pkd2* | 00000034462 | tissue |  | other | Wnt1 cKO: Abnormal pulp cavities, fractured roots | 83 |
| *Postn* | 00000027750 | tissue |  | other | Amelogenesis imperfect and abnormal periodontal ligament | 84 85 |
| *Slc13a5* | 00000020805 | tissue |  | other | Enamel hypoplasia | 86 |
| *Slc39a13* | 00000002105 | tissue |  | other | Dentine hypoplasia | 87 |
| *Sp3* | 00000027109 | tissue |  | transcription factor | Enamel hypoplasia, dentinogenesis imperfecta | 88 |
| *Sppl2a* | 00000027366 | tissue |  | other | Enamel hypoplasia | 89 |
| *Acvr2b* | 00000061393 | double |  | receptor | Double KO (ACVR2A+/-, ACVR2B-/-): Bud stage arrest | 11 |
| *Dlx1* | 00000041911 | double |  | transcription factor | Double KO: Placode stage arrest | 90 |
| *Dlx2* | 00000023391 | double |  | transcription factor | Double KO: Placode stage arrest | 90 |
| *Dlx3* | 00000001510 | double |  | transcription factor | Double KO: Misshaped teeth | 91 |
| *Dlx4* | 00000020871 | double |  | transcription factor | Double KO: Misshaped teeth | 91 |
| *Dlx5* | 00000029755 | double |  | transcription factor | Double KO: Misshaped teeth | 92 |
| *Dlx6* | 00000029754 | double |  | transcription factor | Double KO: Misshaped teeth | 92 |
| *Gli2* | 00000048402 | double |  | transcription factor | Double KO: Initiation stage arrest | 93 |
| *Gli3* | 00000021318 | double |  | transcription factor | Double KO: Initiation stage arrest | 93 |
| *Lhx6* | 00000026890 | double |  | transcription factor | Double KO: Initiation stage arrest | 94 |
| *Lhx8* | 00000096225 | double |  | transcription factor | Double KO: Initiation stage arrest | 94 |
| *Csf1* | 00000014599 | no eruption |  | signal | No eruption of molars | 95 |
| *Fos* | 00000021250 | no eruption |  | transcription factor | No eruption of molars | 96 |
| *Ostm1* | 00000038280 | no eruption |  | other | No eruption of molars | 97 |
| *Pthlh* | 00000048776 | no eruption |  | signal | No eruption of molars | 98 |
| *Tcirg1* | 00000001750 | no eruption |  | other | No eruption of molars | 99 |
| *Traf6* | 00000027164 | no eruption |  | intracellular | No eruption of molars | 100 101 102 |

**References**

1. Matzuk, M. M., Kumar, T. R. & Bradley, A. Different phenotypes for mice deficient in either activins or activin receptor type II. *Nature* **374**, 356-60 (1995).

2. Jia, S. *et al.* Roles of Bmp4 during tooth morphogenesis and sequential tooth formation. *Development* **140**, 423-32 (2013).

3. Dunn, N. R. *et al.* Haploinsufficient Phenotypes inBmp4Heterozygous Null Mice and Modification by Mutations inGli3andAlx4. *Dev. Biol.* **188**, 235-247 (1997).

4. Andl, T. *et al.* Epithelial Bmpr1a regulates differentiation and proliferation in postnatal hair follicles and is essential for tooth development. *Development* **131**, 2257-2268 (2004).

5. Li, C. Y. *et al.* _e-catenin inhibits YAP/TAZ activity to regulate signalling centre formation during tooth development. *Nat. Commun.* **7**, 12133 (2016).

6. Liu, F. *et al.* Wnt/beta-catenin signaling directs multiple stages of tooth morphogenesis. *Dev Biol* **313**, 210-224 (2008).

7. Oommen, S. *et al.* Distinct roles of MicroRNAs in epithelium and mesenchyme during tooth development. *Dev. Dyn.* **241**, 1465-1472 (2012).

8. Michon, F., Tummers, M., Kyyrönen, M., Frilander, M. J. & Thesleff, I. Tooth morphogenesis and ameloblast differentiation are regulated by micro-RNAs. *Dev. Biol.* **340**, 355-368 (2010).

9. De Moerlooze, L. *et al.* An important role for the IIIb isoform of fibroblast growth factor receptor 2 (FGFR2) in mesenchymal-epithelial signalling during mouse organogenesis. *Development* **127**, 483-492 (2000).

10. Matzuk, M. M. *et al.* Functional analysis of activins during mammalian development. *Nature* **374**, 354-357 (1995).

11. Ferguson, C. A. *et al.* The role of effectors of the activin signalling pathway, activin receptors IIA and IIB, and Smad2, in patterning of tooth development. *Development* **128**, 4605-13 (2001).

12. Van Genderen, C. *et al.* Development of several organs that require inductive epithelial- mesenchymal interactions is impaired in LEF-1-deficient mice. *Genes Dev.* **8**, 2691-2703 (1994).

13. Noh, T., Gabet, Y., Cogan, J., Shi, Y. & Tank, A. Lef1 Haploinsufficient Mice Display a Low Turnover and Low Bone Mass Phenotype in a Gender-and Age-Specific Manner. *PLoS One* **4**, 5438 (2009).

14. Satokata, I. & Maas, R. Msx1 deficient mice exhibit cleft palate and abnormalities of craniofacial and tooth development. *Nat. Genet.* **6**, 348-356 (1994).

15. Peters, H., Neubüser, A., Kratochwil, K. & Balling, R. Pax9-deficient mice lack pharyngeal pouch derivatives and teeth and exhibit craniofacial and limb abnormalities. *Genes Dev.* **12**, 2735-2747 (1998).

16. Lin, C. R. *et al.* Pitx2 regulates lung asymmetry, cardiac positioning and pituitary and tooth morphogenesis. *Nature* **401**, 279-282 (1999).

17. Liu, W., Selever, J., Lu, M. F. & Martin, J. F. Genetic dissection of Pitx2 in craniofacial development uncovers new functions in branchial arch morphogenes, late aspects of tooth morphogenesis and cell migration. *Development* **130**, 6375-6385 (2003).

18. Åberg, T. *et al.* Phenotypic Changes in Dentition of Runx2 Homozygote-null Mutant Mice. *J. Histochem. Cytochem.* **52**, 131-139 (2004).

19. Yoda, S., Suda, N., Kitahara, Y., Komori, T. & Ohyama, K. Delayed tooth eruption and suppressed osteoclast number in the eruption pathway of heterozygous Runx2/Cbfa1 knockout mice. *Arch. Oral Biol.* **49**, 435-442 (2004).

20. Dassule, H. R. Shh regulates tooth growth and morphogenesis. **127**, 4775-4785 (2000).

21. Mills, A. A. *et al.* p63 is a p53 homologue required for limb and epidermal morphogenesis. *Nature* **398**, 708-13 (1999).

22. Laurikkala, J. *et al.* P63 Regulates Multiple Signalling Pathways Required for Ectodermal Organogenesis and Differentiation. *Development* **133**, 1553-1563 (2006).

23. Kuraguchi, M. *et al.* Adenomatous polyposis coli (APC) is required for normal development of skin and thymus. *PLoS Genet.* **2**, 1362-1374 (2006).

24. Miletich, I. *et al.* Developmental stalling and organ-autonomous regulation of morphogenesis. *Proc. Natl. Acad. Sci. U. S. A.* **108**, 19270-19275 (2011).

25. Golonzhka, O. *et al.* Ctip2/Bcl11b controls ameloblast formation during mammalian odontogenesis. *Proc. Natl. Acad. Sci. U. S. A.* **106**, 4278-4283 (2009).

26. Katsuragi, Y. *et al.* Bcl11b transcription factor plays a role in the maintenance of the ameloblast-progenitors in mouse adult maxillary incisors. *Mech. Dev.* **130**, 482-492 (2013).

27. Guo, F. *et al.* Bmp2 deletion causes an amelogenesis imperfecta phenotype via regulating enamel gene expression. *J. Cell. Physiol.* **230**, 1871-82 (2015).

28. Feng, J. *et al.* Abnormalities in the enamel in bmp2-deficient mice. *Cells. Tissues. Organs* **194**, 216-21 (2011).

29. Zouvelou, V., Luder, H. U., Mitsiadis, T. A. & Graf, D. Deletion of BMP7 affects the development of bones, teeth, and other ectodermal appendages of the orofacial complex. *J. Exp. Zool. Part B Mol. Dev. Evol.* **312**, 361-374 (2009).

30. Zurowski, C., Jamniczky, H., Graf, D. & Theodor, J. Deletion/loss of bone morphogenetic protein 7 changes tooth morphology and function in Mus musculus: implications for dental evolution in mammals. *R. Soc. open Sci.* **5**, 170761 (2018).

31. Ohazama, A. *et al.* A dual role for Ikk alpha in tooth development. *Dev. Cell* **6**, 219-27 (2004).

32. Srivastava, A. K. *et al.* The Tabby phenotype is caused by mutation in a mouse homologue of the EDA gene that reveals novel mouse and human exons and encodes a protein (ectodysplasin-A) with collagenous domains. *Proc. Natl. Acad. Sci. U. S. A.* **94**, 13069-74 (1997).

33. Tucker, A. S. *et al.* Edar/Eda interactions regulate enamel knot formation in tooth morphogenesis. *Development* **127**, 4691-4700 (2000).

34. Peterkova, R. *et al.* Different morphotypes of the tabby (EDA) dentition in the mouse mandible result from a defect in the mesio-distal segmentation of dental epithelium. *Orthod. Craniofacial Res.* **5**, 215-226 (2002).

35. Charles, C. *et al.* Distinct Impacts of Eda and Edar Loss of Function on the Mouse Dentition. *PLoS One* **4**, (2009).

36. Grüneberg, H. The molars of the tabby mouse, and a test of the 'single-active X-chromosome' hypothesis. *J. Embryol. Exp. Morphol.* **15**, 223-244 (1966).

37. Headon, D. J. *et al.* Gene defect in ectodermal dysplasia implicates a death domain adapter in development. *Nature* **414**, 913-916 (2001).

38. Ruiz-Perez, V. L. *et al.* Evc is a positive mediator of Ihh-regulated bone growth that localises at the base of chondrocyte cilia. *Development* **134**, 2903-2912 (2007).

39. Harada, H. *et al.* FGF10 maintains stem cell compartment in developing mouse incisors. *Development* **129**, 1533-1541 (2002).

40. Yokohama-Tamaki, T. *et al.* Cessation of Fgf10 signaling, resulting in a defective dental epithelial stem cell compartment, leads to the transition from crown to root formation. *Development* **133**, 1359-66 (2006).

41. Häärä, O. *et al.* Ectodysplasin regulates activator-inhibitor balance in murine tooth development through fgf20 signaling. *Dev.* **139**, 3189-3199 (2012).

42. Charles, C. *et al.* Modulation of Fgf3 dosage in mouse and men mirrors evolution of mammalian dentition. *Proc. Natl. Acad. Sci. U. S. A.* **106**, 22364-8 (2009).

43. Jussila, M. *et al.* Suppression of epithelial differentiation by Foxi3 is essential for molar crown patterning. *Development* **142**, 3954-63 (2015).

44. Matzuk, M. M. *et al.* deficient in follistatin. **374**, 354-356 (1995).

45. Wang, X. P. *et al.* Modulation of activin/bone morphogenetic protein signaling by follistatin is required for the morphogenesis of mouse molar teeth. *Dev. Dyn.* **231**, 98-108 (2004).

46. Ohazama, A. *et al.* Primary cilia regulate Shh activity in the control of molar tooth number. *Development* **136**, 897-903 (2009).

47. Mitsiadis, T. A., Graf, D., Luder, H., Gridley, T. & Bluteau, G. BMPs and FGFs target Notch signalling via jagged 2 to regulate tooth morphogenesis and cytodifferentiation. *Development* **137**, 3025-35 (2010).

48. Johnson, E. B., Hammer, R. E. & Herz, J. Abnormal development of the apical ectodermal ridge and polysyndactyly in Megf7-deficient mice. *Hum. Mol. Genet.* **14**, 3523-3538 (2005).

49. Ohazama, A. *et al.* Lrp4 Modulates Extracellular Integration of Cell Signaling Pathways in Development. *PLoS One* **3**, e4092 (2008).

50. Satokata, I. *et al.* Msx2 deficiency in mice causes pleiotropic defects in bone growth and ectodermal organ formation. *Nat. Genet.* **24**, 391-395 (2000).

51. Xu, X., Bringas, P., Soriano, P. & Chai, Y. PDGFR-_ signaling is critical for tooth cusp and palate morphogenesis. *Dev. Dyn.* **232**, 75-84 (2005).

52. Laugel-Haushalter, V. *et al.* RSK2 is a modulator of craniofacial development. *PLoS One* **9**, e84343 (2014).

53. Gritli-Linde, A. *et al.* Shh signaling within the dental epithelium is necessary for cell proliferation, growth and polarization. *Development* **129**, 5323-5337 (2002).

54. Kassai, Y. *et al.* Regulation of mammalian tooth cusp patterning by ectodin. *Science (80-. ).* **309**, 2067-2070 (2005).

55. Nakamura, T. *et al.* Transcription factor epiprofin is essential for tooth morphogenesis by regulating epithelial cell fate and tooth number. *J. Biol. Chem.* **283**, 4825-33 (2008).

56. Klein, O. D. *et al.* Sprouty Genes Control Diastema Tooth Development via Bidirectional Antagonism of Epithelial-Mesenchymal FGF Signaling. *Dev. Cell* **11**, 181-190 (2006).

57. Yang, J. *et al.* Taurodontism, variations in tooth number, and misshapened crowns in Wnt10a null mice and human kindreds. *Mol. Genet. Genomic Med.* **3**, 40-58 (2015).

58. Liu, M., Zhao, S., Lin, Q. & Wang, X.-P. YAP Regulates the Expression of Hoxa1 and Hoxc13 in Mouse and Human Oral and Skin Epithelial Tissues. *Mol. Cell. Biol.* **35**, 1449-1461 (2015).

59. Foster, B. L. *et al.* Tooth root dentin mineralization defects in a mouse model of hypophosphatasia. *J Bone Min. Res* **28**, 271-282 (2013).

60. Fukumoto, S. *et al.* Ameloblastin is a cell adhesion molecule required for maintaining the differentiation state of ameloblasts. *J. Cell Biol.* **167**, 973-983 (2004).

61. Gibson, C. W. *et al.* Amelogenin-deficient Mice Display an Amelogenesis Imperfecta Phenotype. *J. Biol. Chem.* **276**, 31871-31875 (2001).

62. Nakayama, Y., Holcroft, J. & Ganss, B. Enamel hypomineralization and structural defects in amelotin-deficient mice. *J. Dent. Res.* **94**, 697-705 (2015).

63. Biehs, B. *et al.* Bmi1 represses Ink4a/Arf and Hox genes to regulate stem cells in the rodent incisor HHS Public Access. *Nat Cell Biol* **15**, 846-852 (2013).

64. Sun, Y. *et al.* DMP1 processing is essential to dentin and jaw formation. *J. Dent. Res.* **90**, 619-624 (2011).

65. Lu, Y. *et al.* Rescue of odontogenesis in Dmp1-deficient mice by targeted re-expression of DMP1 reveals roles for DMP1 in early odontogenesis and dentin apposition in vivo. *Dev. Biol.* **303**, 191-201 (2007).

66. Feng, J. Q. *et al.* The dentin matrix protein 1 (Dmp1) is specifically expressed in mineralized, but not soft, tissues during development. *J. Dent. Res.* **82**, 776-780 (2003).

67. Sreenath, T. *et al.* Dentin Sialophosphoprotein Knockout Mouse Teeth Display Widened Predentin Zone and Develop Defective Dentin Mineralization Similar to Human Dentinogenesis Imperfecta Type III. *J. Biol. Chem.* **278**, 24874-24880 (2003).

68. Thyagarajan, T., Sreenath, T., Cho, A., Wright, J. T. & Kulkarni, A. B. Reduced Expression of Dentin Sialophosphoprotein Is Associated with Dysplastic Dentin in Mice Overexpressing Transforming Growth Factor-1 in Teeth. *J. Biol. Chem.* **276**, 11016-11020 (2000).

69. Hu, J. C.-C. *et al.* Enamel Defects and Ameloblast-specific Expression in *Enam* Knock-out */lacZ* Knock-in Mice. *J. Biol. Chem.* **283**, 10858-10871 (2008).

70. Zhang, H. *et al.* Generation of Evc2/Limbin global and conditional KO mice and its roles during mineralized tissue formation. *Genesis* **53**, 612-626 (2015).

71. Vogel, P. *et al.* Amelogenesis imperfecta and other biomineralization defects in Fam20a and Fam20c null mice. *Vet. Pathol.* **49**, 998-1017 (2012).

72. Takamori, K. *et al.* Epithelial Fibroblast Growth Factor Receptor 1 Regulates Enamel Formation. *J. Dent. Res.* **87**, 238-243 (2008).

73. de Vicente, J. C. *et al.* Impaired dental cytodifferentiation in Glial cell-line derived growth factor (GDNF) deficient mice. *Ann. Anat. - Anat. Anzeiger* **184**, 85-92 (2002).

74. Vogel, P. *et al.* Malformation of Incisor Teeth in Grem2-/-Mice. *Vet. Pathol.* **52**, 224-229 (2015).

75. Smith, C. E. *et al.* Effect of kallikrein 4 loss on enamel mineralization: comparison with mice lacking matrix metalloproteinase 20. *J. Biol. Chem.* **286**, 18149-18160 (2011).

76. Ryan, M. C., Lee, K., Miyashita, Y. & Carter, W. G. Targeted disruption of the LAMA3 gene in mice reveals abnormalities in survival and late stage differentiation of epithelial cells. *J. Cell Biol.* **145**, 1309-23 (1999).

77. Bartlett, J. D., Zhou, Z., Skobe, Z., Dobeck, J. M. & Tryggvason, K. Delayed Tooth Eruption in Membrane Type-1 Matrix Metalloproteinase Deficient Mice. *Connect. Tissue Res.* **44**, 300-304 (2003).

78. Holmbeck, K. *et al.* MT1-MMP-Deficient Mice Develop Dwarfism, Osteopenia, Arthritis, and Connective Tissue Disease due to Inadequate Collagen Turnover. *Cell* **99**, 81-92 (1999).

79. Caterina, J. J. *et al.* Enamelysin (matrix metalloproteinase 20)-deficient mice display an amelogenesis imperfecta phenotype. *J. Biol. Chem.* **277**, 49598-604 (2002).

80. Xie, F., Dai, Q., Liu, X. & Wang, J. Conditional knockout of conditional raptor/mTORC1 results in dentin malformation. *Front. Physiol.* **10**, 250 (2019).

81. Barron, M. J. *et al.* The cell adhesion molecule nectin-1 is critical for normal enamel formation in mice. *Hum. Mol. Genet.* **17**, 3509-20 (2008).

82. Jheon, A. H. *et al.* PERP regulates enamel formation via effects on cell-cell adhesion and gene expression. *J. Cell Sci.* **124**, 745-54 (2011).

83. Khonsari, R. H. *et al.* Multiple postnatal craniofacial anomalies are characterized by conditional loss of polycystic kidney disease 2 (Pkd2). *Hum. Mol. Genet.* **22**, 1873-1885 (2013).

84. Rios, H. *et al.* Periostin null mice exhibit dwarfism, incisor enamel defects, and an early-onset periodontal disease-like phenotype. *Mol. Cell. Biol.* **25**, 11131-44 (2005).

85. Rios, H. F. *et al.* Periostin is essential for the integrity and function of the periodontal ligament during occlusal loading in mice. *J. Periodontol.* **79**, 1480-90 (2008).

86. Irizarry, A. R. *et al.* Defective enamel and bone development in sodium-dependent citrate transporter (NaCT) Slc13a5 deficient mice. *PLoS One* **12**, e0175465 (2017).

87. Fukada, T. *et al.* The zinc transporter SLC39A13/ZIP13 is required for connective tissue development; its involvement in BMP/TGF-beta signaling pathways. *PLoS One* **3**, e3642 (2008).

88. Bouwman, P. *et al.* Transcription factor Sp3 is essential for post-natal survival and late tooth development. *EMBO J.* **19**, 655-61 (2000).

89. Bronckers, A. L. *et al.* The intramembrane protease SPPL2A is critical for tooth enamel formation. *J. Bone Miner. Res.* **28**, 1622-1630 (2013).

90. Thomas, B. L. *et al.* Role of Dlx-1 and Dlx-2 genes in patterning of the murine dentition. *Development* **124**, 4811-8 (1997).

91. Qiu, M. *et al.* Role of the Dlx homeobox genes in proximodistal patterning of the branchial arches: mutations of Dlx-1, Dlx-2, and Dlx-1 and -2 alter morphogenesis of proximal skeletal and soft tissue structures derived from the first and second arches. *Dev. Biol.* **185**, 165-84 (1997).

92. Beverdam, A. *et al.* Jaw transformation with gain of symmetry after Dlx5/Dlx6 inactivation: mirror of the past? *Genesis* **34**, 221-7 (2002).

93. Hardcastle, Z., Mo, R., Hui, C. C. & Sharpe, P. T. The Shh signalling pathway in tooth development: Defects in Gli2 and Gli3 mutants. *Development* **125**, 2803-2811 (1998).

94. Denaxa, M., Sharpe, P. T. & Pachnis, V. The LIM homeodomain transcription factors Lhx6 and Lhx7 are key regulators of mammalian dentition. *Dev. Biol.* **333**, 324-336 (2009).

95. Fleischmann, A. *et al.* Fra-1 replaces c-Fos-dependent functions in mice. *Genes Dev.* **14**, 2695-700 (2000).

96. Alfaqeeh, S. *et al.* Root and Eruption Defects in *c-Fos* Mice Are Driven by Loss of Osteoclasts. *J. Dent. Res.* **94**, 1724-1731 (2015).

97. Grüneberg, H. Grey-lethal, a new mutation in the house mouse. *J. Hered.* **27**, 105-109 (1936).

98. Philbrick, W. M., Dreyer, B. E., Nakchbandi, I. A. & Karaplis, A. C. Parathyroid hormone-related protein is required for tooth eruption. *Proc. Natl. Acad. Sci. U. S. A.* **95**, 11846-11851 (1998).

99. Bronckers, A. L. J. J. *et al.* Murine ameloblasts are immunonegative for Tcirg1, the v-H-ATPase subunit essential for the osteoclast plasma proton pump. *Bone* **50**, 901-8 (2012).

100. Dickson, K. M., Bhakar, A. L. & Barker, P. A. TRAF6-dependent NF-kB transcriptional activity during mouse development. *Dev. Dyn.* **231**, 122-127 (2004).

101. Lomaga, M. A. *et al.* TRAF6 deficiency results in osteopetrosis and defective interleukin-1, CD40, and LPS signaling. *Genes Dev.* **13**, 1015-24 (1999).

102. Naito, A. *et al.* Severe osteopetrosis, defective interleukin-1 signalling and lymph node organogenesis in TRAF6-deficient mice. *Genes Cells* **4**, 353-62 (1999).

**Table S1.** Genes that are dispensable for mouse tooth development. Published information indicate that although these genes are expressed during mouse molar development, their null mutations have no detectable effects on the tooth phenotype. Only genes having read counts greater than zero in our RNAseq data were included.

| Mouse Ensembl ID | Gene  name | Mouse Ensembl ID | Gene  name | Mouse Ensembl ID | Gene  name |
| --- | --- | --- | --- | --- | --- |
| 00000019256 | *Ahr* | 00000005320 | *Fgfr4* | 00000029838 | *Ptn* |
| 00000022636 | *Alcam* | 00000027004 | *Frzb* | 00000068748 | *Ptprz1* |
| 00000004655 | *Aqp1* | 00000030795 | *Fus* | 00000004768 | *Rab23* |
| 00000028435 | *Aqp3* | 00000022297 | *Fzd6* | 00000042453 | *Reln* |
| 00000024411 | *Aqp4* | 00000025407 | *Gli1* | 00000030110 | *Ret* |
| 00000044217 | *Aqp5* | 00000034220 | *Gpc1* | 00000025158 | *Rfng* |
| 00000032204 | *Aqp9* | 00000029510 | *Gpc2* | 00000070691 | *Runx3* |
| 00000000142 | *Axin2* | 00000048001 | *Hes5* | 00000039656 | *Rxrb* |
| 00000004892 | *Bcan* | 00000064325 | *Hhip* | 00000015843 | *Rxrg* |
| 00000074483 | *Bglap* | 00000078735 | *Il11ra* | 00000020592 | *Sdc1* |
| 00000029335 | *Bmp3* | 00000026638 | *Irf6* | 00000025743 | *Sdc3* |
| 00000032179 | *Bmp5* | 00000001504 | *Irx2* | 00000017009 | *Sdc4* |
| 00000039004 | *Bmp6* | 00000031734 | *Irx3* | 00000057969 | *Sema3b* |
| 00000025217 | *Btrc* | 00000021604 | *Irx4* | 00000034684 | *Sema3f* |
| 00000061048 | *Cdh3* | 00000031738 | *Irx6* | 00000027996 | *Sfrp2* |
| 00000023067 | *Cdkn1a* | 00000022817 | *Itgb5* | 00000025020 | *Slit1* |
| 00000044303 | *Cdkn2a* | 00000019899 | *Lama2* | 00000056427 | *Slit3* |
| 00000022037 | *Clu* | 00000019846 | *Lama4* | 00000045871 | *Slitrk6* |
| 00000001506 | *Col1a1* | 00000029570 | *Lfng* | 00000025323 | *Sp4* |
| 00000031502 | *Col4a1* | 00000023845 | *Lnpep* | 00000056222 | *Spock1* |
| 00000032911 | *Cspg4* | 00000036295 | *Lrrn3* | 00000029304 | *Spp1* |
| 00000032482 | *Cspg5* | 00000027239 | *Mdk* | 00000061762 | *Tac1* |
| 00000062432 | *Cyp26c1* | 00000018169 | *Mfng* | 00000032011 | *Thy1* |
| 00000028519 | *Dab1* | 00000027820 | *Mme* | 00000001131 | *Timp1* |
| 00000019929 | *Dcn* | 00000050578 | *Mmp13* | 00000017466 | *Timp2* |
| 00000026131 | *Dst* | 00000043613 | *Mmp3* | 00000030516 | *Tjp1* |
| 00000028017 | *Egf* | 00000017737 | *Mmp9* | 00000034917 | *Tjp3* |
| 00000038418 | *Egr1* | 00000019982 | *Myb* | 00000025215 | *Tlx1* |
| 00000028289 | *Epha7* | 00000005125 | *Ndrg1* | 00000028364 | *Tnc* |
| 00000006369 | *Fbln1* | 00000048616 | *Nog* | 00000060548 | *Tnfrsf19* |
| 00000064080 | *Fbln2* | 00000038146 | *Notch3* | 00000026875 | *Traf1* |
| 00000036585 | *Fgf1* | 00000025969 | *Nrp2* | 00000022996 | *Wnt10b* |
| 00000037225 | *Fgf2* | 00000032855 | *Pkd1* |  |  |
| 00000027208 | *Fgf7* | 00000028681 | *Ptch2* |  |  |

**Table S2.** Descriptive statistics of the expression of different gene categories. Developmental-process category is based on Gene ontology 0032502 criteria and Other-category includes all the remaining protein coding genes.

| Embryonic  Day/Platform | Gene  category | *n* | Median | Mean | Range | SD |
| --- | --- | --- | --- | --- | --- | --- |
| **Microarray** |  |  |  |  |  |  |
| E13 mouse | Progression | 15 | 7.867 | 8.094 | 6.73–9.71 | 0.787 |
|  | Shape | 28 | 7.347 | 7.087 | 4.30–9.14 | 1.249 |
|  | Double | 10 | 7.419 | 7.360 | 4.84–8.87 | 1.082 |
|  | Tissue | 27 | 6.359 | 6.231 | 3.70–10.61 | 1.771 |
|  | Dispensable | 98 | 6.599 | 6.501 | 3.56–9.78 | 1.440 |
|  | Dev. process | 3983 | 6.100 | 6.063 | 1.45–10.43 | 1.468 |
|  | Other | 14825 | 5.616 | 5.601 | 1.45–12.02 | 1.570 |
| E14 mouse | Progression | 15 | 8.029 | 8.087 | 7.11–9.81 | 0.749 |
|  | Shape | 28 | 7.592 | 7.389 | 4.63–9.71 | 1.124 |
|  | Double | 10 | 7.143 | 7.035 | 5.12–8.06 | 0.807 |
|  | Tissue | 27 | 6.343 | 6.315 | 3.81–10.24 | 1.713 |
|  | Dispensable | 98 | 6.798 | 6.606 | 3.75–9.29 | 1.360 |
|  | Dev. process | 3983 | 6.109 | 6.067 | 1.47–10.71 | 1.460 |
|  | Other | 14825 | 5.604 | 5.577 | 1.53–11.72 | 1.563 |
| **RNAseq** |  |  |  |  |  |  |
| E13 mouse | Progression | 15 | 12.125 | 11.665 | 7.07–14.26 | 1.639 |
|  | Shape | 28 | 9.695 | 9.522 | 3.82–13.19 | 2.443 |
|  | Double | 10 | 10.752 | 10.391 | 5.17–12.83 | 2.099 |
|  | Tissue | 27 | 9.463 | 7.619 | -2.25–12.41 | 4.838 |
|  | Dispensable | 100 | 9.031 | 8.556 | -2.35–13.31 | 3.569 |
|  | Dev. process | 4106 | 8.658 | 7.331 | -2.36–13.47 | 4.203 |
|  | Other | 16165 | 7.033 | 5.571 | -2.36–13.87 | 4.610 |
| E14 mouse | Progression | 15 | 12.150 | 12.022 | 9.78–14.32 | 1.150 |
|  | Shape | 28 | 10.133 | 10.053 | 4.92–13.01 | 1.913 |
|  | Double | 10 | 10.949 | 10.647 | 6.67–12.86 | 1.727 |
|  | Tissue | 27 | 9.691 | 7.735 | -2.25–12.47 | 4.865 |
|  | Dispensable | 100 | 9.541 | 8.861 | -2.35–13.74 | 3.566 |
|  | Dev. process | 4106 | 8.708 | 7.383 | -2.36–13.55 | 4.217 |
|  | Other | 16165 | 7.062 | 5.591 | -2.36–14.06 | 4.620 |
| E16 mouse | Progression | 15 | 12.147 | 12.134 | 10.10–14.27 | 1.024 |
|  | Shape | 28 | 10.310 | 10.239 | 4.17–12.92 | 1.899 |
|  | Double | 10 | 11.351 | 10.733 | 8.10–12.73 | 1.479 |
|  | Tissue | 27 | 9.917 | 7.842 | -2.25–12.25 | 4.950 |
|  | Dispensable | 100 | 9.562 | 9.015 | -2.35–14.40 | 3.539 |
|  | Dev. process | 4106 | 8.772 | 7.419 | -2.36–13.63 | 4.200 |
|  | Other | 16165 | 7.193 | 5.634 | -2.36–13.90 | 4.612 |
| E15 rat | Progression | 15 | 11.184 | 11.290 | 6.92–13.90 | 1.643 |
|  | Shape | 28 | 9.510 | 9.372 | 3.88–13.34 | 2.141 |
|  | Double | 10 | 10.083 | 9.712 | 4.58–12.25 | 2.263 |
|  | Tissue | 27 | 8.972 | 7.554 | -1.19–14.35 | 4.556 |
|  | Dispensable | 95 | 8.691 | 8.652 | -1.50–16.96 | 3.491 |
|  | Dev. process | 3843 | 8.488 | 7.252 | -2.35–16.38 | 4.071 |
|  | Other | 12473 | 7.701 | 6.254 | -2.35–15.25 | 4.212 |
| E17 rat | Progression | 15 | 11.505 | 11.444 | 8.96–14.03 | 1.243 |
|  | Shape | 28 | 9.763 | 9.723 | 4.10–13.29 | 1.932 |
|  | Double | 10 | 9.921 | 9.462 | 4.69–11.84 | 2.154 |
|  | Tissue | 27 | 9.565 | 7.581 | -1.19–14.57 | 4.634 |
|  | Dispensable | 95 | 9.007 | 8.859 | -1.50–17.26 | 3.450 |
|  | Dev. process | 3843 | 8.575 | 7.297 | -2.35–16.58 | 4.068 |
|  | Other | 12473 | 7.739 | 6.290 | -2.35–15.30 | 4.212 |

**Table S3.** Permutation tests for microarray, RNAseq and scRNAseq expression levels of progression, shape, and double categories compared to tissue, dispensable, developmental process, and other-gene categories. These are permutation tests of differences between group medians (*p*-values of one-tailed significance levels obtained using 10 000 permutations).

| Embryonic  Day/Patform | Gene  category | Tissue | Dispensable | Developmental  process | Other |
| --- | --- | --- | --- | --- | --- |
| **Microarray** |  |  |  |  |  |
| E13 mouse | Progression | 0.0098 | 0.0426 | 0.0003 | 0.0000 |
|  | Shape | 0.0689 | 0.0901 | 0.0010 | 0.0000 |
|  | Double | 0.0978 | 0.1945 | 0.0322 | 0.0039 |
| E14 mouse | Progression | 0.0028 | 0.0466 | 0.0000 | 0.0000 |
|  | Shape | 0.0027 | 0.0345 | 0.0001 | 0.0000 |
|  | Double | 0.1743 | 0.5010 | 0.1072 | 0.0132 |
| **RNAseq** |  |  |  |  |  |
| E13 mouse | Progression | 0.0379 | 0.0059 | 0.0370 | 0.0565 |
|  | Shape | 0.8391 | 0.5138 | 0.2468 | 0.1082 |
|  | Double | 0.3008 | 0.2425 | 0.1637 | 0.1533 |
| E14 mouse | Progression | 0.0199 | 0.0299 | 0.0339 | 0.0608 |
|  | Shape | 0.6456 | 0.4646 | 0.1211 | 0.0759 |
|  | Double | 0.3229 | 0.2751 | 0.1425 | 0.1416 |
| E16 mouse | Progression | 0.0240 | 0.0302 | 0.0312 | 0.0649 |
|  | Shape | 0.5935 | 0.3100 | 0.0886 | 0.0770 |
|  | Double | 0.3436 | 0.1464 | 0.1040 | 0.1349 |
| E15 rat | Progression | 0.0451 | 0.0513 | 0.0493 | 0.0426 |
|  | Shape | 0.5185 | 0.4038 | 0.2286 | 0.0780 |
|  | Double | 0.4984 | 0.3395 | 0.2507 | 0.1524 |
| E17 rat | Progression | 0.0443 | 0.0242 | 0.0382 | 0.0335 |
|  | Shape | 0.6503 | 0.3759 | 0.1520 | 0.0505 |
|  | Double | 0.7371 | 0.5851 | 0.3048 | 0.1677 |
| **scRNAseq** |  |  |  |  |  |
| E14 mouse | Progression | 0.0146 | 0.0071 | 0.0310 | 0.0295 |
|  | Shape | 0.9968 | 0.9087 | 0.7788 | 0.1357 |
|  | Double | 0.1233 | 0.0683 | 0.1262 | 0.0939 |

**Table S4.** Pathway genes used to compare expression levels. Genes marked to belong into more than one pathway were tabulated only once in the calculations. The FGF pathway includes also EGF pathway genes. LG = ligand, RC = receptor, IC = intracellular cofactor, TF = transcription factor.

| Ensembl ID | Gene name | Pathway | Type | Ensembl ID | Gene name | Pathway | Type |
| --- | --- | --- | --- | --- | --- | --- | --- |
| 00000026836 | *Acvr1* | TGFB | RC | 00000024913 | *Lrp5* | Wnt | IC |
| 00000026834 | *Acvr1c* | TGFB | RC | 00000030201 | *Lrp6* | Wnt | IC |
| 00000052155 | *Acvr2a* | TGFB | RC | 00000050567 | *Maml1* | Notch | TF |
| 00000061393 | *Acvr2b* | TGFB | RC | 00000031925 | *Maml2* | Notch | TF |
| 00000054693 | *Adam10* | Notch | IC | 00000061143 | *Maml3* | Notch | TF |
| 00000052593 | *Adam17* | Notch | IC | 00000004936 | *Map2k1* | FGF | IC |
| 00000001729 | *Akt1* | FGF/TGFB | IC | 00000035027 | *Map2k2* | FGF | IC |
| 00000004056 | *Akt2* | FGF/TGFB | IC | 00000018932 | *Map2k3* | TGFB | IC |
| 00000035262 | *Amh* | TGFB | LG | 00000033352 | *Map2k4* | TGFB | IC |
| 00000005871 | *Apc* | Wnt | IC | 00000020623 | *Map2k6* | TGFB | IC |
| 00000020135 | *Apc2* | Wnt | IC | 00000028284 | *Map3k7* | EDA /TGFB | IC |
| 00000015750 | *Aph1a* | Notch | IC | 00000063358 | *Mapk1* | FGF/TGFB | IC |
| 00000001127 | *Araf* | FGF/TGFB | IC | 00000053436 | *Mapk14* | TGFB | IC |
| 00000022911 | *Arl13b* | Hh | IC | 00000063065 | *Mapk3* | FGF/TGFB | IC |
| 00000018909 | *Arrb1* | Hh | IC | 00000021936 | *Mapk8* | TGFB | IC |
| 00000060216 | *Arrb2* | Hh | IC | 00000020366 | *Mapk9* | TGFB | IC |
| 00000024182 | *Axin1* | Wnt | IC | 00000018169 | *Mfng* | Notch | IC |
| 00000000142 | *Axin2* | Wnt | IC | 00000024294 | *Mib1* | Notch | IC |
| 00000030046 | *Bmp10* | TGFB | LG | 00000029060 | *Mib2* | Notch | IC |
| 00000023279 | *Bmp15* | TGFB | LG | 00000034121 | *Mks1* | Hh | IC |
| 00000027358 | *Bmp2* | TGFB | LG | 00000028991 | *Mtor* | FGF | IC |
| 00000029335 | *Bmp3* | TGFB | LG | 00000018501 | *Ncor1* | Notch | IC |
| 00000021835 | *Bmp4* | TGFB | LG | 00000029478 | *Ncor2* | Notch | IC |
| 00000032179 | *Bmp5* | TGFB | LG | 00000003458 | *Ncstn* | Notch | IC |
| 00000039004 | *Bmp6* | TGFB | LG | 00000006435 | *Neurl1a* | Notch | IC |
| 00000008999 | *Bmp7* | TGFB | LG | 00000034413 | *Neurl1b* | Notch | IC |
| 00000032726 | *Bmp8a* | TGFB | LG | 00000028163 | *Nfkb1* | EDA/FGF/TGFB | TF |
| 00000002384 | *Bmp8b* | TGFB | LG | 00000025225 | *Nfkb2* | EDA/FGF/TGFB | TF |
| 00000021796 | *Bmpr1a* | TGFB | RC | 00000021025 | *Nfkbia* | EDA | TF |
| 00000052430 | *Bmpr1b* | TGFB | RC | 00000031661 | *Nkd1* | Wnt | IC |
| 00000067336 | *Bmpr2* | TGFB | RC | 00000021567 | *Nkd2* | Wnt | IC |
| 00000022687 | *Boc* | Hh | RC | 00000037171 | *Nodal* | TGFB | LG |
| 00000002413 | *Braf* | FGF/TGFB | IC | 00000026923 | *Notch1* | Notch | RC |
| 00000025217 | *Btrc* | Hh | IC | 00000027878 | *Notch2* | Notch | RC |
| 00000038119 | *Cdon* | Hh | RC | 00000038146 | *Notch3* | Notch | RC |
| 00000025199 | *Chuk* | EDA | IC | 00000015468 | *Notch4* | Notch | RC |
| 00000024576 | *Csnk1a1* | Hh/Wnt | IC | 00000027852 | *Nras* | FGF/TGFB | IC |
| 00000025162 | *Csnk1d* | Wnt | IC | 00000021224 | *Numb* | Notch | IC |
| 00000022433 | *Csnk1e* | Wnt | IC | 00000063160 | *Numbl* | Notch | IC |
| 00000032384 | *Csnk1g1* | Hh/Wnt | IC | 00000040586 | *Ofd1* | Hh | IC |
| 00000003345 | *Csnk1g2* | Hh/Wnt | IC | 00000029231 | *Pdgfra* | FGF | RC |
| 00000073563 | *Csnk1g3* | Hh/Wnt | IC | 00000027665 | *Pik3ca* | FGF/TGFB | IC |
| 00000006932 | *Ctnnb1* | Wnt | IC | 00000031834 | *Pik3r2* | FGF/TGFB | IC |
| 00000023000 | *Dhh* | Hh | LG | 00000016933 | *Plcg1* | FGF | IC |
| 00000024868 | *Dkk1* | Wnt | IC | 00000031169 | *Porcn* | Wnt | IC |
| 00000028031 | *Dkk2* | Wnt | IC | 00000005469 | *Prkaca* | Hh | IC |
| 00000030772 | *Dkk3* | Wnt | IC | 00000050965 | *Prkca* | FGF | IC |
| 00000031535 | *Dkk4* | Wnt | IC | 00000019969 | *Psen1* | Notch | IC |
| 00000014773 | *Dll1* | Notch | LG | 00000010609 | *Psen2* | Notch | IC |
| 00000003436 | *Dll3* | Notch | LG | 00000021466 | *Ptch1* | Hh | RC |
| 00000027314 | *Dll4* | Notch | LG | 00000028681 | *Ptch2* | Hh | RC |
| 00000029603 | *Dtx1* | Notch | IC | 00000013663 | *Pten* | FGF | IC |
| 00000029071 | *Dvl1* | Wnt | IC | 00000000441 | *Raf1* | FGF/TGFB | IC |
| 00000020888 | *Dvl2* | Wnt | IC | 00000039191 | *Rbpj* | Notch | TF |
| 00000003233 | *Dvl3* | Wnt | IC | 00000024927 | *Rela* | EDA | TF |
| 00000047193 | *Dync2h1* | Hh | IC | 00000002983 | *Relb* | EDA | TF |
| 00000059327 | *Eda* | EDA | LG | 00000025158 | *Rfng* | Notch | IC |
| 00000034457 | *Eda2r* | EDA | RC | 00000007815 | *Rhoa* | TGFB | IC |
| 00000003227 | *Edar* | EDA | RC | 00000034177 | *Rnf43* | Wnt | IC |
| 00000095105 | *Edaradd* | EDA | IC | 00000024290 | *Rock1* | TGFB | IC |
| 00000028017 | *Egf* | FGF | LG | 00000057132 | *Rpgrip1* | Hh | IC |
| 00000020122 | *Egfr* | FGF | RC | 00000031309 | *Rps6ka3* | FGF | IC |
| 00000029122 | *Evc* | Hh | IC | 00000031548 | *Sfrp1* | Wnt | IC |
| 00000028086 | *Fbxw7* | Notch | IC | 00000027996 | *Sfrp2* | Wnt | IC |
| 00000036585 | *Fgf1* | FGF | LG | 00000021319 | *Sfrp4* | Wnt | IC |
| 00000021732 | *Fgf10* | FGF | LG | 00000018822 | *Sfrp5* | Wnt | IC |
| 00000042826 | *Fgf11* | FGF | LG | 00000042626 | *Shc1* | FGF | IC |
| 00000022523 | *Fgf12* | FGF | LG | 00000020312 | *Shc2* | FGF | IC |
| 00000031137 | *Fgf13* | FGF | LG | 00000002633 | *Shh* | Hh | LG |
| 00000025551 | *Fgf14* | FGF | LG | 00000029050 | *Ski* | Hh | IC |
| 00000031230 | *Fgf16* | FGF | LG | 00000031681 | *Smad1* | TGFB | TF |
| 00000037225 | *Fgf2* | FGF | LG | 00000024563 | *Smad2* | TGFB | TF |
| 00000031603 | *Fgf20* | FGF | LG | 00000032402 | *Smad3* | TGFB | TF |
| 00000020327 | *Fgf22* | FGF | LG | 00000024515 | *Smad4* | TGFB | TF |
| 00000031074 | *Fgf3* | FGF | LG | 00000021540 | *Smad5* | TGFB | TF |
| 00000050917 | *Fgf4* | FGF | LG | 00000027796 | *Smad9* | TGFB | TF |
| 00000029337 | *Fgf5* | FGF | LG | 00000001761 | *Smo* | Hh | RC |
| 00000000183 | *Fgf6* | FGF | LG | 00000038037 | *Socs1* | FGF | IC |
| 00000027208 | *Fgf7* | FGF | LG | 00000020027 | *Socs2* | FGF | IC |
| 00000021974 | *Fgf9* | FGF | LG | 00000053113 | *Socs3* | FGF | IC |
| 00000031565 | *Fgfr1* | FGF | RC | 00000024241 | *Sos1* | FGF | IC |
| 00000030849 | *Fgfr2* | FGF | RC | 00000034801 | *Sos2* | FGF | IC |
| 00000054252 | *Fgfr3* | FGF | RC | 00000036169 | *Sostdc1* | TGFB/Wnt | LG |
| 00000005320 | *Fgfr4* | FGF | RC | 00000022114 | *Spry2* | FGF | IC |
| 00000020170 | *Frs2* | FGF | IC | 00000024427 | *Spry4* | FGF | IC |
| 00000021765 | *Fst* | TGFB | LG | 00000026104 | *Stat1* | FGF | TF |
| 00000011658 | *Fuz* | Hh | IC | 00000040033 | *Stat2* | FGF | TF |
| 00000044674 | *Fzd1* | Wnt | RC | 00000004040 | *Stat3* | FGF | TF |
| 00000081683 | *Fzd10* | Wnt | RC | 00000062939 | *Stat4* | FGF | TF |
| 00000050288 | *Fzd2* | Wnt | RC | 00000004043 | *Stat5a* | FGF | TF |
| 00000007989 | *Fzd3* | Wnt | RC | 00000020919 | *Stat5b* | FGF | TF |
| 00000049791 | *Fzd4* | Wnt | RC | 00000002147 | *Stat6* | FGF | TF |
| 00000045005 | *Fzd5* | Wnt | RC | 00000028718 | *Stil* | Hh | IC |
| 00000022297 | *Fzd6* | Wnt | RC | 00000025231 | *Sufu* | Hh | IC |
| 00000041075 | *Fzd7* | Wnt | RC | 00000034601 | *Ta3* | Hh | IC |
| 00000036904 | *Fzd8* | Wnt | RC | 00000015755 | *Tab2* | EDA | IC |
| 00000049551 | *Fzd9* | Wnt | RC | 00000000782 | *Tcf7* | Wnt | TF |
| 00000031714 | *Gab1* | FGF | IC | 00000055799 | *Tcf7l1* | Wnt | TF |
| 00000052957 | *Gas1* | Hh | IC | 00000024985 | *Tcf7l2* | Wnt | TF |
| 00000109523 | *Gdf1* | TGFB | LG | 00000038593 | *Tctn1* | Hh | IC |
| 00000025352 | *Gdf11* | TGFB | LG | 00000002603 | *Tgfb1* | TGFB | LG |
| 00000025407 | *Gli1* | Hh | TF | 00000039239 | *Tgfb2* | TGFB | LG |
| 00000048402 | *Gli2* | Hh | TF | 00000021253 | *Tgfb3* | TGFB | LG |
| 00000021318 | *Gli3* | Hh | TF | 00000007613 | *Tgfbr1* | TGFB | RC |
| 00000059923 | *Grb2* | FGF | IC | 00000032440 | *Tgfbr2* | TGFB | RC |
| 00000050069 | *Grem2* | TGFB | LG | 00000029287 | *Tgfbr3* | TGFB | RC |
| 00000024858 | *Grk2* | Hh | IC | 00000008305 | *Tle1* | Wnt | TF |
| 00000057177 | *Gsk3a* | Wnt | IC | 00000032280 | *Tle3* | Wnt | TF |
| 00000022812 | *Gsk3b* | Hh/Wnt | IC | 00000026875 | *Traf1* | EDA | IC |
| 00000022528 | *Hes1* | Notch | TF | 00000026942 | *Traf2* | EDA | IC |
| 00000025499 | *Hras* | FGF/TGFB | IC | 00000021277 | *Traf3* | EDA | IC |
| 00000038564 | *Ift172* | Hh | IC | 00000017386 | *Traf4* | EDA /TGFB | IC |
| 00000017858 | *Ift52* | Hh | IC | 00000027164 | *Traf6* | EDA/TGFB | IC |
| 00000032965 | *Ift57* | Hh | IC | 00000028173 | *Wls* | Wnt | TF |
| 00000040040 | *Ift88* | Hh | IC | 00000022997 | *Wnt1* | Wnt | LG |
| 00000006538 | *Ihh* | Hh | LG | 00000026167 | *Wnt10a* | Wnt | LG |
| 00000031537 | *Ikbkb* | EDA | IC | 00000022996 | *Wnt10b* | Wnt | LG |
| 00000004221 | *Ikbkg* | EDA | IC | 00000015957 | *Wnt11* | Wnt | LG |
| 00000041324 | *Inhba* | TGFB | LG | 00000029671 | *Wnt16* | Wnt | LG |
| 00000037035 | *Inhbb* | TGFB | LG | 00000010797 | *Wnt2* | Wnt | LG |
| 00000060798 | *Intu* | Hh | IC | 00000027840 | *Wnt2b* | Wnt | LG |
| 00000027598 | *Itch* | Notch | IC | 00000000125 | *Wnt3* | Wnt | LG |
| 00000027276 | *Jag1* | Notch | LG | 00000009900 | *Wnt3a* | Wnt | LG |
| 00000002799 | *Jag2* | Notch | LG | 00000036856 | *Wnt4* | Wnt | LG |
| 00000028530 | *Jak1* | FGF | IC | 00000021994 | *Wnt5a* | Wnt | LG |
| 00000024789 | *Jak2* | FGF | IC | 00000030170 | *Wnt5b* | Wnt | LG |
| 00000031805 | *Jak3* | FGF | IC | 00000033227 | *Wnt6* | Wnt | LG |
| 00000052684 | *Jun* | TGFB | TF | 00000030093 | *Wnt7a* | Wnt | LG |
| 00000046731 | *Kctd11* | Hh | IC | 00000022382 | *Wnt7b* | Wnt | LG |
| 00000018395 | *Kif3a* | Hh | IC | 00000012282 | *Wnt8a* | Wnt | LG |
| 00000050382 | *Kif7* | Hh | IC | 00000036961 | *Wnt8b* | Wnt | LG |
| 00000030265 | *Kras* | FGF/TGFB | IC | 00000000126 | *Wnt9a* | Wnt | LG |
| 00000027985 | *Lef1* | Wnt | TF | 00000018486 | *Wnt9b* | Wnt | LG |
| 00000029570 | *Lfng* | Notch | IC | 00000041961 | *Znrf3* | Wnt | LG |
| 00000027253 | *Lrp4* | Wnt | RC |  |  |  |  |

**Table S5.** Descriptive statistics of the number of cells in which different gene types are expressed. The number of analyzed cells was 30930 for E14 mouse scRNAseq.

| Type | *n* | Median | Mean | Range | SD |
| --- | --- | --- | --- | --- | --- |
| **Progression and shape category genes** | | |  |  |  |
| Ligand | 13 | 4585.0 | 7765.77 | 386–25360 | 7440.31 |
| Receptor | 7 | 13879.0 | 12897.14 | 607–23912 | 8261.00 |
| Transcription factor | 11 | 14165.0 | 13215.91 | 531–24359 | 8816.32 |
| Intracellular | 12 | 14522.0 | 15296.67 | 4247–30260 | 8741.45 |
| **Progression and shape category pathway genes** | | | |  |  |
| Ligand | 65 | 2308.0 | 4020.12 | 2–25360 | 5211.06 |
| Receptor | 38 | 8071.5 | 9437.92 | 101–27666 | 7831.50 |
| Transcription factor | 34 | 12832.0 | 12492.38 | 290–28213 | 8142.03 |
| Intracellular | 129 | 11919.0 | 12944.92 | 474–30770 | 8014.32 |
